# The Application of the Neuroprotective and Potential Antioxidant Effect of Ergotamine Mediated by Targeting N-Methyl-D-Aspartate Receptors

**DOI:** 10.3390/antiox11081471

**Published:** 2022-07-28

**Authors:** Shinhui Lee, Sanung Eom, Khoa V. A. Nguyen, Jiwon Lee, Youngseo Park, Hye Duck Yeom, Junho H. Lee

**Affiliations:** 1 Department of Biotechnology, Chonnam National University, Gwangju 61186, Korea; dltlstn39@jnu.ac.kr (S.L.); 178291@jnu.ac.kr (S.E.); khvn@jnu.ac.kr (K.V.A.N.); lizy1@jnu.ac.kr (J.L.); 0seo28@jnu.ac.kr (Y.P.); 2 GoPath Laboratories, Buffalo Grove, IL 60089, USA

**Keywords:** NMDA, N-methyl-D-aspartate, ergotamine, ergot alkaloid, antioxidant, neuronal disease, two-electrode voltage clamp, free reactive oxygen species

## Abstract

(1) Background: The N-methyl-D-aspartate receptors (NMDARs) mediate fast excitatory currents leading to depolarization. Postsynaptic NMDARs are ionotropic glutamate receptors that mediate excitatory glutamate or glycine signaling in the CNS and play a primary role in long-term potentiation, which is a major form of use-dependent synaptic plasticity. The overstimulation of NMDARs mediates excessive Ca^2+^ influx to postsynaptic neurons and facilitates more production of ROS, which induces neuronal apoptosis. (2) Methods: To confirm the induced inward currents by the coapplication of glutamate and ergotamine on NMDARs, a two-electrode voltage clamp (TEVC) was conducted. The ergotamine-mediated inhibitory effects of NR1a/NR2A subunits were explored among four different kinds of recombinant NMDA subunits. In silico docking modeling was performed to confirm the main binding site of ergotamine. (3) Results: The ergotamine-mediated inhibitory effect on the NR1a/NR2A subunits has concentration-dependent, reversible, and voltage-independent properties. The major binding sites were V169 of the NR1a subunit and N466 of the NR2A subunit. (4) Conclusion: Ergotamine effectively inhibited NR1a/NR2A subunit among the subtypes of NMDAR. This inhibition effect can prevent excessive Ca^2+^ influx, which prevents neuronal death.

## 1. Introduction

The N-methyl-D-aspartate (NMDA) receptor (NMDAR), which mediates fast-acting excitatory currents leading to depolarization, is a tetrameric ionotropic channel belonging to the ligand-gated glutamate receptor family. The pore blockade by Mg^2+^ is decreased with increased neuronal activity, and the permeation of the NMDAR pore is stimulated to enable activation and allow the influx of Ca^2+^ and Na^+^ into neurons; however, the NMDAR is largely inactive when the cell membrane is at resting potential [1]. NMDAR activity is induced by: (1) binding of active compounds such as neurotransmitters, for example, agonists (glutamate) or other co-agonists (D-serine or glycine), and (2) agents causing membrane depolarization, which impact the channel pore blockade by Zn^2+^ or Mg^2+^. NMDARs are classified into three groups based on sequence homology, namely glycine/D-serine-binding NR1, glutamate-binding NR2 subunits (NR2A, NR2B, NR2C, and NR2D), and glycine-binding NR3 (NR3A and NR3B) subunits [2,3,4]. All NMDARs have the same membrane topology: one large extracellular N-terminal domain, four transmembrane domains (M1, M2, M3, and M4; M2 encodes the re-entrant pore [approximately 150 amino acids long]), one intracellular cytoplasmic C-terminal domain (which can interact with structural, adaptor, and scaffolding proteins), and one extracellular ligand-binding domain [5,6,7,8].

Presynaptic NMDARs play pivotal roles in excitatory neurotransmission and synaptic plasticity. They facilitate presynaptic neurotransmitter release and modulate mechanisms controlling synaptic maturation and plasticity during the formative periods of brain development. In addition to the role of NMDARs in the excitatory synapse, its impact on synaptic plasticity as a result of postsynaptic and presynaptic NMDAR activity is one of the most striking effects on the central nervous system (CNS) [9,10]. Postsynaptic NMDARs are ionotropic glutamate receptors that mediate excitatory glutamate or glycine signaling in the CNS. Postsynaptic NMDARs play a primary role in long-term potentiation (LTP), a significant form of use-dependent synaptic plasticity [11]. Postsynaptic NMDARs are associated with various neuropathological symptoms and are, therefore, an attractive target as a therapeutic agent for neurological disorders such as Alzheimer’s disease (AD) [12]. Ca^2+^ cannot enter the cell at resting membrane potential owing to Mg^2+^ blockage; however, the blockage is disappeared on depolarization of the postsynaptic membrane, leading to the initiation of the Ca^2+^ influx into the cell. Ca^2+^ plays a role in increasing synaptic sensitivity as a secondary messenger; however, an overstimulation of NMDARs induces an influx of excess Ca^2+^ and triggers neuronal apoptosis due to toxic metabolic processes [13].

The intracellular Ca^2+^ concentration is a critical factor for the survival of cells. Ca^2+^ signaling is involved in a variety of physiological functions, including nerve excitability, cell migration, growth, muscle contraction, and synaptic plasticity (learning and memory) [14,15]. Several cellular functions (tricarboxylic acid cycle, ATP production, and reactive oxygen species [ROS] production) depend on mitochondrial Ca^2+^ signaling [14,16]. Ca^2+^ transport into the mitochondrial matrix plays an essential role in ROS generation and redox signaling; therefore, Ca^2+^ homeostasis is tightly controlled. NMDAR-dependent Ca^2+^ influx is essential for LTP, a memory-enhanced activity [17].

**Figure 1:**
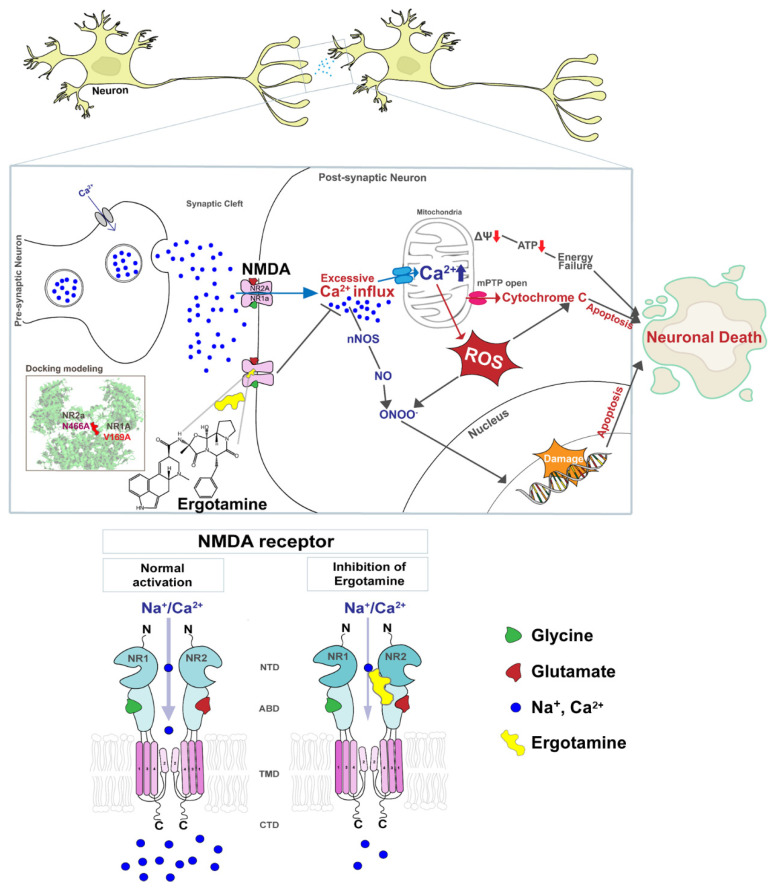
Schematic diagram of the NMDA receptor and excessive Ca^2+^ ions influx induces cellular apoptosis in the event of the overstimulation of this receptor. The overstimulation of NMDAR increases the intracellular Ca^2+^ influx, thus increasing ROS accumulation, damaging genetic material, and ultimately leading to apoptosis. Ergotamine derived from ergot alkaloids can modulate intracellular Ca^2+^ concentration by inhibiting hyperstimulation to NMDAR.

However, excessive Ca^2+^ accumulation induces the opening of the mitochondrial permeability transition pore, membrane depolarization, excessive accumulation of ROS, and release of cytochrome c. Increased ROS generation and cytochrome c release activate pro-apoptotic factors that lead to cell death [18,19,20]. Figure 1 presents a schematic diagram that NMDARs overstimulation leads to the excessive influx of intracellular Ca^2+^ that affects the mitochondrial metabolism, redox status, and DNA damage response and induces apoptosis, but the ergotamine can inhibit the overstimulation. Therefore, the overactivation of NMDARs induces failure of redox regulation as a result of excessive Ca^2+^ accumulation and mitochondrial dysfunction, as well as induces neuronal loss through apoptosis, which may lead to neuropathological disease development.

Ergot alkaloids are considered a pharmacological treasure as they possess pharmacological properties, such as a high affinity for various receptors. Small modifications in ergot alkaloids can yield new compounds with various pharmacological effects [21]. Ergotamine, one such ergot alkaloid, initially known as an antimigraine drug, has been used as a treatment for severe migraine for over 50 years [22].

In this study, ergotamine was identified as a subtype-selective antagonist or modulator that could suppress glutamate-evoked current on these four target subunits of NMDARs. The molecular mechanism of its involvement was realized by voltage-clamp technology and mutagenesis studies by comparing various mutation complexes. The best-fit docking conformation between ergotamine and NR1a/NR2A subunits at a potential site was suggested. In addition, ergotamine inhibits NMDAR activity, eliminating excessive Ca^2+^ influx and modulating redox regulation, and prevents neuronal apoptosis. This result indicates that ergotamine may lead to neuroprotective effects.

## 2. Materials and Methods

### 2.1. Materials

**Figure 2:**
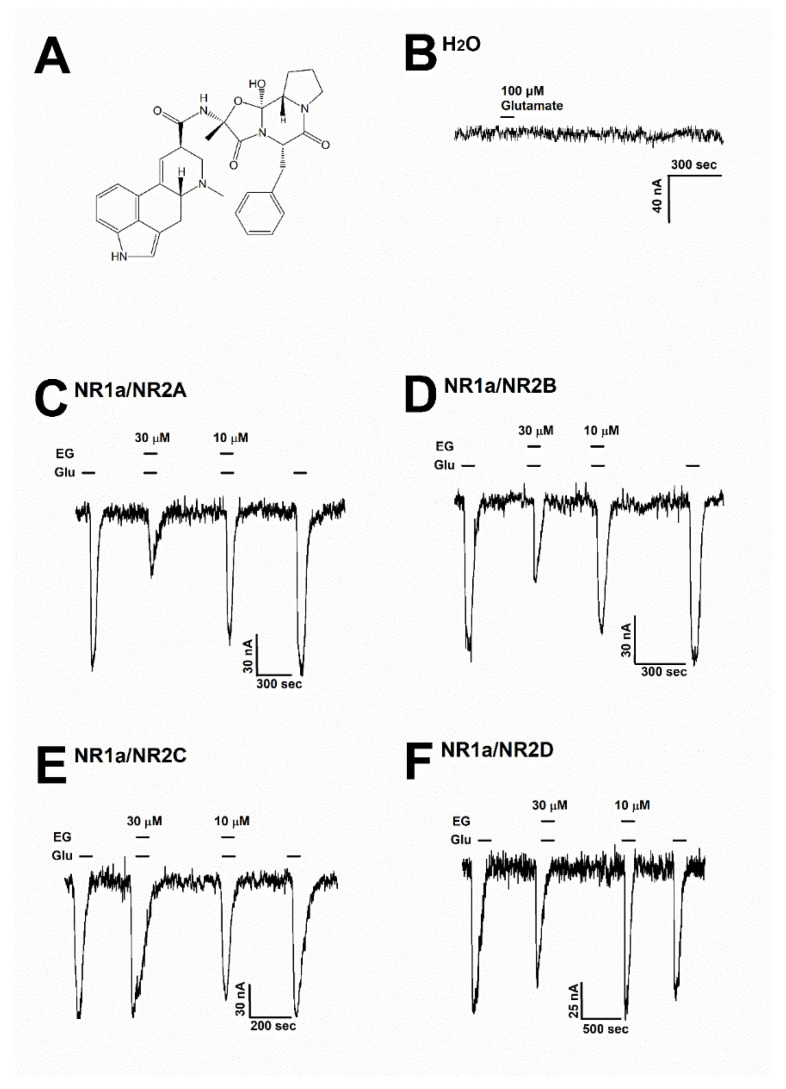
(**A**) The chemical structure of ergotamine. (**B**) The H_2_O-injected oocytes did not cause any change with the treatment of 100 µM glutamate (*n* = 6–8 oocytes from four different frogs). (**C**–**F**) Glutamate induced inward currents with or without ergotamine (30 µM and 10 µM). For each subunit of NMDARs, the responses after treating with either glutamate (100 µM) alone or together with ergotamine (30 and 10 µM). Voltage clamp recording was conducted at a holding potential of −80 mV. The coapplication of ergotamine with glutamate resulted in modulation of the recombinant receptors (**C**) NR1a/NR2A, (**D**) NR1a/NR2B, (**E**) NR1a/NR2C, and (**F**) NR1a/NR2D, which in turn reduced glutamate-evoked inward current in a reversible manner (*n* = 6–8 oocytes from four different frogs).

Ergotamine (Wuchan Chem Faces Biochemical, Wuhan, China) was dissolved in a dimethyl sulfoxide solvent and diluted to prepare a recording solution for the next experiment (less than 0.03% DMSO in the final recording solution). Figure 2A shows the chemical structure of ergotamine. The mouse NMDAR subunit cDNAs included the NR1 (GenBank accession number: MR225704), NR2A (MR227135), NR2B (MR227077), NR2C (MR222676), and NR2D (MR220972) subunits, which were purchased from OriGene (Rockville, MD, USA). All other compounds were supplied by Merck (Darmstadt, Germany).

### 2.2. Preparation of Xenopus Oocytes and Microinjection

According to the Chonnam National University guidelines for animal care (CNU IACUC-YB-2016-07), *X. laevis* frogs were cared for and handled in adherence to the Korean Xenopus Resource Center for Research (KXRCR000001) manual. Surgery was performed to manually collect the *X. laevis* oocytes. The selected oocytes were isolated by shaking incubation in Ringer solution (82.5 mM NaCl, 2 mM KCl, 1 mM MgCl_2_, 5 mM HEPES; pH 7.4) supplemented with 0.5 μg/μL of collagenase within 2 h and then maintained in ND96 incubation solution (96 mM NaCl, 2 mM KCl, 1.8 mM CaCl_2_, 1 mM MgCl_2_, 5 mM HEPES, 2.5 mM sodium pyruvate, 50 mg/mL gentamicin solution; pH 7.4) at 16 °C. All the incubation solutions were changed daily. Aliquots of 40 nL mRNA solutions were prepared, and the mRNAs were pulled with a glass capillary tubing (15–20 µm in diameter) by using a 10 nL nanoinjector (VWR Scientific, Seattle, WA, USA). The electrophysiological experiments were performed within 3–5 days of the oocyte isolation.

### 2.3. NR1a/NR2A Receptor Mutation and In Vitro Transcription of cDNAs

MAX QuikChange mutagenesis kits (Stratagene, CA, USA) were used for mutating NMDAR subunits prior to amplification by PCR. The success of PCR was evaluated using DNA sequencing analysis by Cosmo Gentech Inc. (Seoul, Seongdong-gu, Korea) after the PCR product was transfected into XL1-Blue supercompetent cells, followed by screening. The identified DNA was linearized using the restriction enzyme NotI, and then transcribed into RNA using T7 in vitro transcription kits (Ambion, Austin, TX, USA). The final RNA products were resuspended in diethyl pyrocarbonate and stored as aliquots at a final concentration of 1 μg/µL at −80 °C for the next experiment.

### 2.4. Molecular Docking Study with Three-Dimensional (3D) Modeling

For the molecular docking study of the NMDAR and ergotamine interaction, the protein structure was obtained from the Protein Data Bank (PDB); the PDB ID of the selected protein structure was 7EOS. The 3D structure of ergotamine was referenced in PubChem (CID code: 8223). The docking study was performed in a basic setting using AutoDock Tools (Scripps Research Institute [version 4.2.6], La Jolla, CA, USA). The performance state of the protein was enhanced by removing water molecules from the macromolecule, adding polarity and hydrogen ions, and computing the Gasteiger charges. The models were selected on the basis of intermolecular energy, inhibition constant, binding structures, and binding energy. The complex of ergotamine and NMDAR (NR1a/NR2A subunits) was analyzed using LIGPLOT, which calculated the binding activity between ergotamine and NMDAR. The distance between the NMDAR and ergotamine molecule interaction site was measured using PyMol.

### 2.5. Data Recording

An oocyte clamp (OC-725C; Warner Instruments, Hamden, CT, USA) with a perfusion chamber was used for the two-electrode voltage-clamp recordings at room temperature. A recording solution (ND96 bath solution) was prepared as described previously [23] and applied with ergotamine and glutamate during recording, according to the experiment design. Oocytes were placed into the chamber with the ND96 bath solution, flowing at a rate of 1 mL/min. Two electrodes filled with 3M KCl (electrolyte solution, 0.2–0.7 MΩ resistance) were stabbed at a random position in every oocyte. Experiments were set with a −80 mV holding potential for the current recording and −100 to +60 mV within 300 ms for ramping the voltage relationship of NMDARs. All the data were collected and analyzed using Digidata 1320 (Molecular Devices, Sunnyvale, CA, USA) and pCLAMP 9 software (Axon Instruments, Union City, CA, USA).

### 2.6. Data Analysis

To investigate the concentration–response curves of the role of ergotamine on glutamate-stimulated inward current (I_Glu_), various concentrations of ergotamine were examined to measure I_Glu_ by voltage-clamp recording in the presence of glutamate. Origin Pro 8.0 (Origin, Northampton, MA, USA) was used to describe the relationship between the concentration of ergotamine and percent inhibition of I_Glu_ by ergotamine, based on the Hill equation described as: V_min_ + (V_max_ − V_min_) × [x]^n^/([IC_50_]^n^ + [x]^n^), where y is the peak current at a given concentration of ergotamine, V_max_ is the maximal peak value, IC_50_ is the half-maximal inhibitory concentration of ergotamine on I_Glu_, [x] is the concentration of ergotamine and glutamate, and n is the interaction coefficient. All the values are presented as the standard error of the mean. The differences between the means of the control and application values were determined using one-way ANOVA with a Tukey test. *p* <0.01 was considered statistically significant.

## 3. Results

### 3.1. Inhibitory Effect of Ergotamine on Various N-Methyl-D-Aspartate Receptors

To assess the impact of ergotamine on various NMDAR subunits, 100 µM glutamate was supplemented into a bath solution to stimulate a large inward current in oocytes that were injected with NMDAR subunit (NR1a/NR2A, NR1a/NR2B, NR1a/NR2C, and NR1a/NR2D) mRNAs. Figure 2B shows that H_2_O-injected oocytes were not affected by the application of glutamate (100 µM). The effects of ergotamine (30 and 10 µM) on glutamate (100 µM)-evoked current are shown in Figure 2C–F. The inhibition response of I_Glu_ is caused by the application of (30 µM) ergotamine in the presence of glutamate (100 µM) on each type of NMDAR subunit (NR1a/2A, NR1a/2B, NR1a/2C, and NR1a/2D) was recorded at 65.2 ± 6.5%, 45.5 ± 8.5%, 10.2 ± 4.3%, and 18.6 ± 5.3%, respectively. The coapplication of ergotamine with glutamate (100 µM) had nearly no impact on I_Glu_ on NR1a/2C and NR1a/2D subunits. On the NR1a/2B subunits, the coapplication of ergotamine with glutamate could only slightly reduce the intensity of the currents elicited by glutamate compared to treatment with glutamate only; however, it markedly inhibited I_Glu_ on the NR1a/2A subunit receptor in the same condition. These results implied that the coapplication of ergotamine on inward currents of NR1a/2A subunits has an inhibitory manner, and the inhibition was reversible.

### 3.2. Ergotamine Concentration-Dependent Inhibition of I_Glu_

Previous data showed the inhibitory effect of ergotamine on the application of NMDA subunit (NR1a/2A and NR1a/2B) receptors. Different concentrations of ergotamine, ranging from 1–100 μM, and different potencies for I_Glu_ were analyzed. The results demonstrated that ergotamine-mediated inhibition of glutamate-induced inward currents for NR1a/2A and NR1a/2B subunits was concentration-dependent. As shown in Table 1, the IC_50_ values for each recombinant NR1a/NR2A–D were 15.3 ± 6.1, 15.7 ± 4.8, 6.5 ± 3.6, and 13.8 ± 6.4 µM, respectively; the values of the Hill coefficient for recombinant NR1a/NR2A–D were 2.1 ± 1.5, 2.1 ± 1.0, 0.8 ± 0.5, and 1.2 ± 0.6, respectively, corroborating previous analysis results. In summary, the potent inhibitory effect of ergotamine on glutamate-evoked currents on the NR1a/NR2A subunits was highest compared to that on other NMDAR subunits. A further study on the NR1a/NR2A subunits focused on clarifying the role of the inhibition of ergotamine in NMDARs is required.

### 3.3. In Silico Docking of Ergotamine to Wild-Type and Mutant NR1a/NR2A Subunits

**Figure 3:**
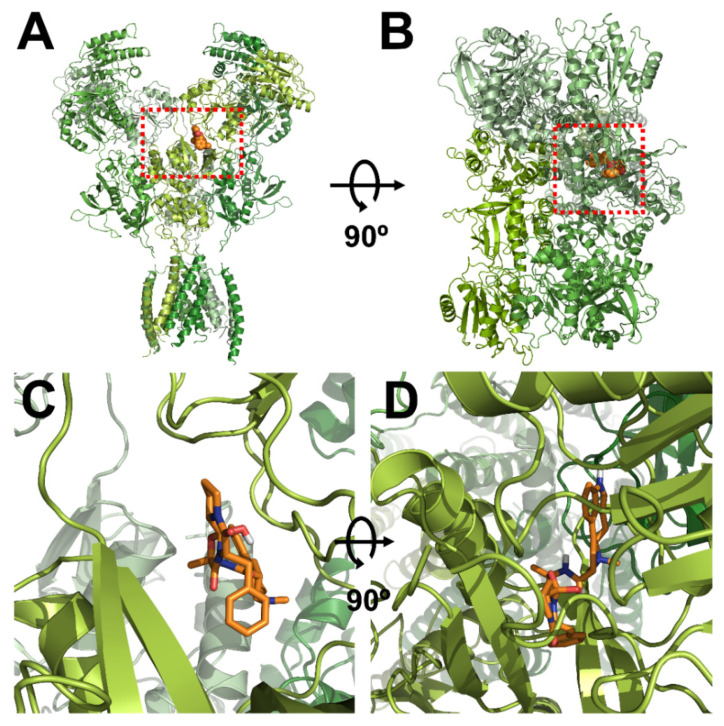
Computational molecular modeling of ergotamine docked to the human NR1a/NR2A receptor. (**A**,**C**) Side views of the docked ergotamine complex with NMDA channel. (**B**,**D**) Binding pocket and docking results of ergotamine and NMDA channel, respectively.

**Figure 4:**
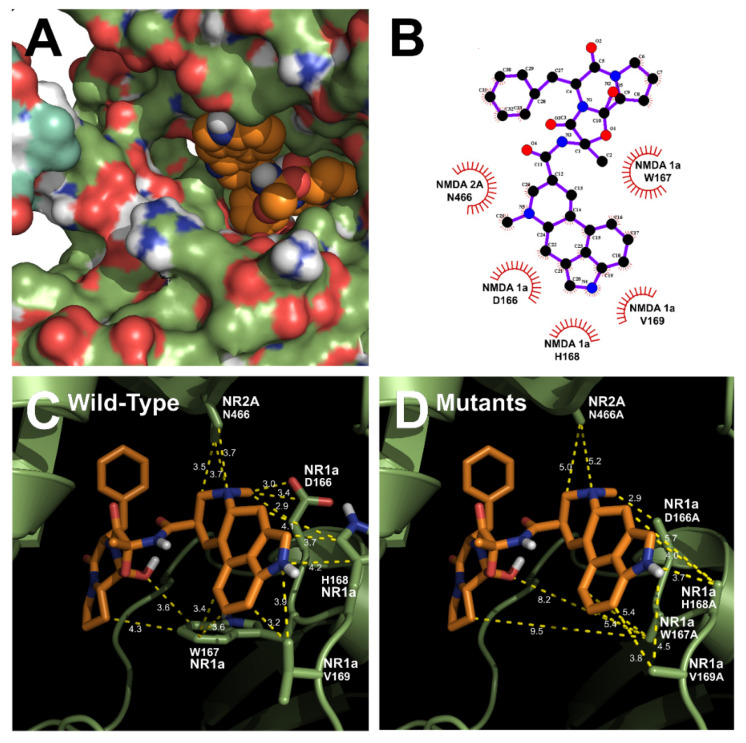
Predicted binding mode of ergotamine and all the favorable interactions with several residues in the active site of the human NR1a/NR2A receptor. (**A**,**B**) Interaction between ergotamine and wild-type NR1a/NR2A. (**C**) Residues in wild-type NR1a/NR2A receptor interacting with the ergotamine molecule. (**D**) Change in the interaction distance of ergotamine in mutant-type NR1a/NR2A receptor. Based on the change in this distance, the residue that directly interacts with ergotamine was identified.

The homology models of wild-type NR1a/NR2A subunits and mutant channels were built to predict the possible ligand-binding site of ergotamine on the NR1a/NR2A subunits. The in silico docking of ergotamine to wild-type and mutant models of the subunit was analyzed. Using the docking tool AutoDock, we predicted the binding affinity of ergotamine to NR1a/NR2A subunits based on the binding free energy of each site. The values of all the binding free energies are shown in Table 2. The best-fit docking was noted as NR1a W167, NR1a H168, NR1a V169, NR2A P435, and NR2A N466 (lowest energy pose) at −7.45 kcal/mol with intermolecular energy at −9.54 kcal/mol. Other complexes with a docking score of −6.45 kcal/mol showed a strong interaction with intermolecular energy that was higher than that for the above-predicted complexes. The values of the root-mean-square deviation (RMSD) were insignificantly dissimilar between the two predicted groups (complex #1 and #2) and lower than 0.25 nm, indicating the binding state stability of the protein. The lowest docking score included binding energy, intermolecular energy, and RMSD at 0.21 (complex #1), indicating that the change of predicting conformation is favorable and a near-native pose. The following residues were constructed as a binding site (with active radium 2.1 Å): W167, H168, and V169 residues in NR1a subunit; and P435 and N466 residues in NR2A subunit. Virtual screening and computational molecular conformation of ergotamine docked to the NR1a/NR2A channels were performed. The pose was generated and then evaluated, as shown in Figure 3 and Figure 4, respectively. Thus, ergotamine could not only interact with three residues (W167, H168, and V169) on the NR1a subunit but also bind to the two residues (P435 and N466) on the NR2A subunit. According to these results, the in silico docking model suggests that these residues, which mediate the inhibition of I_Glu_, may allow ergotamine to interact with specific amino acids of NR1a/NR2A subunits via hydrogen bond formation.

### 3.4. Effect of Ergotamine in Various NR1a/NR2A Subunit Mutations

**Figure 5:**
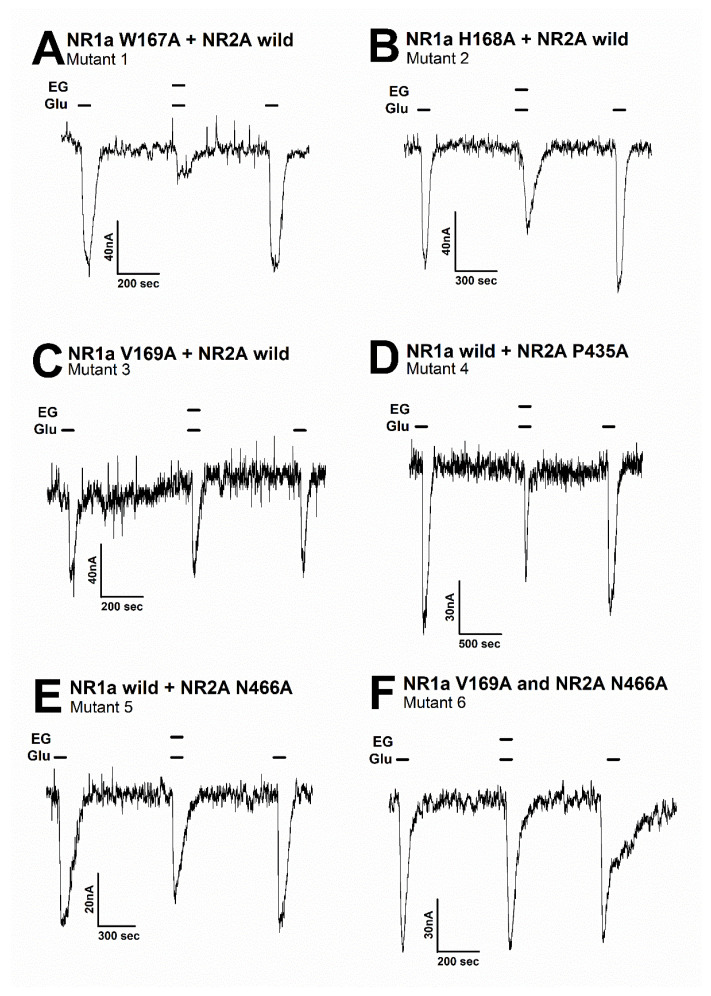
The inward current of several mutant types on the glutamate-evoked current of NR1a/NR2A receptor with or without ergotamine. Representative traces of current induced by application of glutamate (100 µM) alone or together with ergotamine (100 µM) for various mutants: (**A**) mutant 1 (NR1a subunit W167A and NR2A wild-type), (**B**) mutant 2 (NR1a subunit H168A and NR2A wild-type), (**C**) mutant 3 (NR1a subunit V169A and NR2A wild-type), (**D**) mutant 4 (NR1a wild-type and NR2A subunit P435A), (**E**) mutant 5 (NR1a wild-type and NR2A subunit N466A), and (**F**) mutant 6 (double mutant-type NR1a subunit V169A and NR2A subunit N466A). Experiments were performed separately, and data were collected from several oocytes (*n* = 6–8 oocytes from four different frogs).

To validate the best near-native pose for the selected residues from the NR1a/NR2A subunits with ergotamine, each residue was mutated into alanine to elucidate its functional role. Thus, the inhibitory effect of ergotamine on the I_Glu_ current on oocytes expressing these mutants was further analyzed to determine whether ergotamine interacted with the channel on these residues as the binding site or not. The values of the inhibitory response of ergotamine on I_Glu_ for each mutant type are shown in Table 3. According to the results of the predicted mutants, the representative inward current traces of various mutant types with or without ergotamine are shown in Figure 5. A single mutation in each subunit receptor seems to exert effects on the inhibition of ergotamine (63.7% ± 3.5% to 32.7% ± 6.8%).

**Figure 6:**
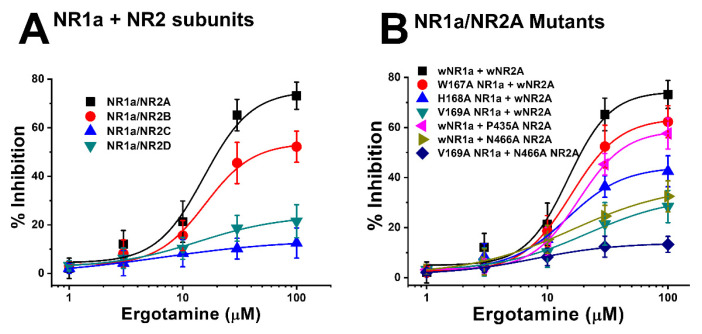
(**A**) Concentration-response curves for the effect of ergotamine on expressed NMDA receptors. The percentage inhibition by ergotamine was calculated based on the average of peak inward current elicited by glutamate and that of peak inward current elicited by glutamate and ergotamine. Each point represents the mean ± S.E.M. (*n* = 6–8 oocytes from four different frogs). (**B**) Concentration–response curves for the inhibition of ergotamine on glutamate-induced inward current of NR1a/NR2A mutant types. NR1a (W167A, H168A, and V169A) and NR2A mutants (P435A and N466A) were cross-combined between wild-type/mutants of each subunit. Ergotamine reduced I_Glu_ in a concentration-dependent manner in the wild-type. Experiments were performed at a holding potential of −80 mV. The value of IC_50_, I_max_, and n_H_ (Hill coefficient) are shown in Table 3. Each point represents the mean ± S.E.M. (*n* = 6–8 oocytes from four different frogs).

Moreover, the mutation in mutant 1 (Figure 5A) (NR1a W167A + NR2A wild-type) showed almost no difference in the response of glutamate-stimulated current compared with the response to the wild-type NR1a/NR2A subunits, indicating that the W167A residue has no functional role in the inhibition of ergotamine. Notably, mutant 6 (Figure 5F) (NR1a V169A and NR2A N466A) strongly eliminated the inhibitory ability of ergotamine at approximately 13.9% ± 0.7%. According to the ergotamine-induced inhibition curves in wild-type (Figure 6A), the I_max_ values for the NMDAR subunits NR1a/NR2A, NR1a/NR2B, NR1a/NR2D, and NR1a/NR2C were recorded at 75.2% ± 9.7%, 53.7% ± 5.8%, 24.0% ± 5.1%, and 13.9% ± 3.0%, respectively. To compare these results with mutant-type, the inhibition percentage of ergotamine applied to each mutation type was expressed as a sigmoid curve using the Hill equation, and the results are shown in Figure 6B. The inhibitory responses of ergotamine on the oocytes expressing the double-mutant type were recorded with an I_max_ value of 13.9% ± 0.7% and IC_50_ of 7.8 ± 1.1 µM. The Hill coefficient (n_H_) of 1.3 ± 0.2 are shown in Table 3. Thus, ergotamine could interact with both the residues, namely NR1a V169 and NR2A N466 of the NR1a/NR2A subunits.

## 4. Discussion

Various neuropathological diseases begin with the neuronal loss [24]. Neuronal death is caused by (1) beta-amyloid, (2) glutamate, (3) FeSO_4_, (4) haloperidol, (5) H_2_O_2_, and (6) ischemic damage. Several factors are involved in neuronal loss; however, neuronal loss can also be caused by ROS [25,26]. ROS are byproducts generated initially in cellular physiology and are involved in host defense, redox signaling, hormone biosynthesis, mitogenesis, and oxygen sensing as a result of neuronal signal transduction [27]. Excessive intracellular Ca^2+^ accumulation results in the overproduction of ROS owing to a partial reduction of oxygen by mitochondrial electron transport chains, cytoplasmic lipoxygenase, and peroxisome flavoprotein oxidases. The balance of detoxification and generation of ROS induces them to play their original role. The transient generation of localized ROS plays an important role in receptor-mediated cellular signaling [16,28]. However, when the redox system is disrupted, Ca^2+^ regulation is affected, leading to oxidative stress caused by excess free radicals, which is associated with various diseases [29]. Therefore, maintenance of intracellular Ca^2+^ at an optimal concentration is essential.

Neuropathological disorders caused by ROS overproduction include Huntington’s disease, Parkinson’s disease, and AD. The pathogenesis for AD was hypothesized as follows: brain cell death due to cytotoxicity is caused by the accumulation of beta-amyloid. Aggregates of beta-amyloid are found in AD patients in the form of peptides ranging from 39 to 43 amino acids in length [30]. These aggregates directly induce oxidative stress in the cell by interacting with the nerve cell membrane and stimulating ROS accumulation, thereby inducing neurotoxicity. The accumulation of beta-amyloid also makes (1) the cells more susceptible to glutamate, (2) interferes with intracellular Ca^2+^ regulation, and (3) interacts with various neurotransmitter receptors [25].

Recently, it has been shown that neuronal cell dysfunction and oxidative cell death are related to AD-associated beta-amyloid protein accumulation. Therefore, the importance of intracellular redox regulation is emphasized. Neuronal depolarization is induced when the activity of the respiratory enzyme deteriorates owing to aging, the ROS scavenging ability decreases, and the energy metabolism process is impaired. Neuronal depolarization induces activation of NMDARs in the brain, and excessive Ca^2+^ accumulation in the cell increases ROS generation [29]. These hypotheses can be linked, suggesting that the accumulation of beta-amyloid (cause of AD) induces neurotoxicity, making it more susceptible to toxins such as glutamate. In addition, beta-amyloid accumulation disrupts the regulation of intracellular Ca^2+^. As a result, beta-amyloid aggregates interact with nerve cell membranes and induce the accumulation of ROS in the cells [25]. Therefore, although the beta-amyloid hypothesis is representative of AD, the free radical theory due to aging is also accepted. Nerve cells are extremely sensitive to oxidation, and antioxidant drugs are emerging as a new alternative for the prevention and treatment of neurodegenerative diseases. However, available drugs are very limited, and although treatment using antioxidants is an emerging alternative, studies supporting this research are lacking.

NMDARs are involved in many diseases, including neurodegenerative and psychiatric disorders. NMDAR subunits exhibit different functions depending on their combination. The subunit NR1 can assemble in pairwise combinations with one of four possible NR2 subunits (di-heteromeric NMDARs) or can assemble as a complex of NR2 and NR3 (tri-heteromeric NMDARs) [31,32,33,34]. This fact explains why various NMDARs that comprise one of the four different NR2 subunits were reported as having separate pharmacological and functional characteristics in the brain. The decrease in expression of various NMDAR subunits during senescence strongly implies they play a crucial role in memory, cognition, synaptic transmission, and, particularly, spatial information [35,36,37]. Several neuropathology reports have revealed that glutamate excitotoxicity and Ca^2+^ overload are associated and lead to neuronal death and synaptic dysfunction. Moreover, these reviews reported the role of NMDARs in aggravating chronic diseases, especially AD and Parkinson’s disease [38,39]. It was confirmed that extra-synaptic NMDARs with glutamatergic transmission are associated with other neurological diseases such as schizophrenia, Huntington’s disease, major depressive disorder, ischemia/reperfusion injury, amyotrophic lateral sclerosis, and stroke.

The expression of NR2A and NR2B subunits, found mostly in the cerebral cortex and hippocampus, was reported to be involved in the impairment of several functions of the brain, including cognitive performance and synaptic function [40,41,42,43]. The impact of neuronal damage on cerebral ischemia by inducing neuronal death and survival has been confirmed to involve NR2A-containing receptors [44]. In particular, NR2A-containing receptors were reported to regulate neurodegeneration in rats with hyperhomocysteinemia. Experiments on NR2A-containing receptor-knockout mice proved that NR2A mediated its pharmacological action by inhibiting ischemia-induced functional deficits [10]. In addition, the modulation of drug self-administration memory in the infralimbic medial prefrontal cortex by the NR2A-containing receptor suggests its potential as a therapeutic to reduce relapse possibility [45].

Consequently, the development of NMDAR-selective antagonists proves its immense therapeutic potential. Although ergotamine is an ergot alkaloid known to exhibit high efficacy in cardiovascular disease and migraine treatment [21], its neuroprotective effect owing to the interaction with NMDARs is unknown. Ergotamine is a drug that has been used in the past; however, its ability to cross the blood–brain barrier remains unclear. Additionally, its beneficial effect on headaches was inferred, not proved. Through in vitro porcine brain capillary endothelial cell experiments, it was confirmed that an effective concentration of ergotamine could penetrate the blood-brain barrier, owing to its exceptional transport properties, and could effectively activate nerve cells [46]; the toxicity caused by ergotamine association must also be considered. Furthermore, it was confirmed that ergotamine can regulate intracellular Ca^2+^ concentration by inhibiting NR1a/NR2A subunits in vitro, and this result suggests that ergotamine may regulate redox status and may be a potential treatment for several neurological diseases.

## 5. Conclusions

The ergotamine-mediated inhibitory effects of NR1a/NR2A subunits were explored among four different kinds of recombinant NMDA subunits. The ergotamine-mediated inhibitory effect on the current evoked by glutamate on oocytes expressing NR1a/NR2A subunits was concentration-dependent and reversible and worked as voltage-independent. The pharmacological mechanism of ergotamine-mediated suppression of I_Glu_ in cells expressing NR1a/NR2A subunits was clarified. The results of double mutations of V169 of the NR1a subunit and N466 of the NR2A subunit showed that both residues are important binding sites for ergotamine. The potential mechanisms by which ergotamine blocks the channel were visualized by performing homology modeling and ligand docking to the transmembrane domain of the NR1a/NR2A subunits. Excessive activity of NMDARs induces excessive Ca^2+^ influx into cells and may lead to neuronal death. These results suggest that the inhibition of NMDAR by ergotamine may have a neuroprotective function. Through in vitro molecular study and in silico docking modeling, ergotamine effectively inhibited NR1a/NR2A, a subtype of NMDAR, and it was found that it could be a drug that can potentially modulate redox status by regulating Ca^2+^ influx into the neurons.

## Figures and Tables

**Table 1:** The value of I_max_, IC_50_, and n_H_ (Hill coefficient) of ergotamine for the glutamate-evoked current in each recombinant receptor. Values represent means ± S.E.M. (*n* = 6–8/group). IC_50_, Hill’s coefficient; I_max_ value as determined as described in Materials and methods.

	I_max_	IC_50_	n_H_
NR1a/NR2A	75.2 ± 9.7	15.3 ± 6.1	2.1 ± 1.5
NR1a/NR2B	53.7 ± 5.8	15.7 ± 4.8	2.1 ± 1.0
NR1a/NR2C	13.9 ± 3.0	6.5 ± 3.6	0.8 ± 0.5
NR1a/NR2D	24.0 ± 5.1	13.8 ± 6.4	1.2 ± 0.6

**Table 2:** The predicted docking sites and binding energy of NR1a/NR2A receptor and ergotamine.

	Binding Energy	KI (mM)	Intermole-Cular Energy	Internal Energy	RMSD * (nm)	Binding Residues (Amino Acids)
#1	–7.54	0.15	–9.54	–1.21	0.21	NR1a W167, NR1a H168, NR2A V169, NR2A P435, NR2A N466
#2	–6.54	1.01	–6.54	–1.01	0.19	NR1a N210, NR1a G215, NR2A N216, NR2A C510, NR2A E551
#3	–5.44	1.24	–5.25	–1.20	0.19	NR1a K155, NR1a H156, NR1a G773, NR2A N523, NR2A R767
#4	–4.57	1.98	–4.25	–1.32	0.18	NR1a E528, NR2A T433, NR2A V434, NR2A S468

* RMSD (root-mean-square deviation of atomic positions). Binding energy, intermolecular energy, and internal energy (kcal/mol).

**Table 3:** Effect of ergotamine on the glutamate-evoked current of wild-type NR1a/NR2A receptor and its various mutants.

No.	Mutants	I_max_	IC_50_	n_H_
	NR1a wild + NR2A wild	74.4 ± 8.4	14.9 ± 4.7	2.5 ± 1.4
1	NR1a W167A + NR2A wild	63.7 ± 3.5	15.7 ± 2.0	2.2 ± 0.5
2	NR1a H168A + NR2A wild	44.3 ± 5.9	14.6 ± 4.0	1.9 ± 0.8
3	NR1a V169A + NR2A wild	32.7 ± 6.8	21.4 ± 10.7	1.2 ± 0.5
4	NR1a wild + NR2A P435A	58.8 ± 0.9	18.2 ± 0.5	2.3 ± 0.1
5	NR1a wild + NR2A N466A	37.2 ± 2.2	16.1 ± 2.0	1.0 ± 0.1
6	NR1a V169A + NR2A N466A	13.9 ± 0.7	7.8 ± 1.1	1.3 ± 0.2

Values represent means ± S.E.M. (*n* = 6–8/group).

## Data Availability

Data is contained within the article.

## References

[1] Nicoll R.A., Roche K.W. (2013). Long-term potentiation: Peeling the onion. Neuropharmacology.

[2] Traynelis S.F., Wollmuth L.P., McBain C.J., Menniti F.S., Vance K.M., Ogden K.K., Hansen K.B., Yuan H., Myers S.J., Dingledine R. (2010). Glutamate receptor ion channels: Structure, regulation, and function. Pharmacol. Rev..

[3] Paoletti P., Bellone C., Zhou Q. (2013). NMDA receptor subunit diversity: Impact on receptor properties, synaptic plasticity and disease. Nat. Rev. Neurosci..

[4] Smothers C.T., Woodward J.J. (2007). Pharmacological characterization of glycine-activated currents in HEK 293 cells expressing N-methyl-D-aspartate NR1 and NR3 subunits. J. Pharmacol. Exp. Ther..

[5] Masuko T., Kashiwagi K., Kuno T., Nguyen N.D., Pahk A.J., Fukuchi J.I., Igarashi K., Williams K. (1999). A regulatory domain (R1–R2) in the amino terminus of theN-methyl-D-aspartate receptor: Effects of spermine, protons, and ifenprodil, and structural similarity to bacterial leucine/isoleucine/valine binding protein. Mol. Pharmacol..

[6] Karakas E., Simorowski N., Furukawa H. (2009). Structure of the zinc-bound amino-terminal domain of the NMDA receptor NR2B subunit. EMBO J..

[7] Karakas E., Simorowski N., Furukawa H. (2011). Subunit arrangement and phenylethanolamine binding in GluN1/GluN2B NMDA receptors. Nature.

[8] Romero-Hernandez A., Furukawa H. (2017). Novel mode of antagonist binding in NMDA receptors revealed by the crystal structure of the GluN1-GluN2A ligand-binding domain complexed to NVP-AAM077. Mol. Pharmacol..

[9] Slikker W., Paule M.G., Wang C. (2018). Handbook of Developmental Neurotoxicology.

[10] Banerjee A., Larsen R., Philpot B.D., Paulsen O. (2016). Roles of presynaptic NMDA receptors in neurotransmission and plasticity. Trends Neurosci..

[11] Deutschenbaur L., Beck J., Kiyhankhadiv A., Mühlhauser M., Borgwardt S., Walter M., Hasler G., Sollberger D., Lang U.E. (2016). Role of calcium, glutamate and NMDA in major depression and therapeutic application. Prog. Neuro-Psychopharmacol. Biol. Psychiatry.

[12] Hedegaard M., Hansen K.B., Andersen K.T., Bräuner-Osborne H., Traynelis S.F. (2012). Molecular pharmacology of human NMDA receptors. Neurochem. Int..

[13] Duchen M.R. (2012). Mitochondria, calcium-dependent neuronal death and neurodegenerative disease. Pflügers Arch.-Eur. J. Physiol..

[14] Delierneux C., Kouba S., Shanmughapriya S., Potier-Cartereau M., Trebak M., Hempel N. (2020). Mitochondrial calcium regulation of redox signaling in cancer. Cells.

[15] Godfraind T. (2005). Antioxidant effects and the therapeutic mode of action of calcium channel blockers in hypertension and atherosclerosis. Philos. Trans. R. Soc. B Biol. Sci..

[16] Chernorudskiy A.L., Zito E. (2017). Regulation of calcium homeostasis by ER redox: A close-up of the ER/mitochondria connection. J. Mol. Biol..

[17] Sibarov D., Antonov S. (2018). Calcium-dependent desensitization of NMDA receptors. Biochemistry.

[18] Mammucari C., Raffaello A., Reane D.V., Gherardi G., De Mario A., Rizzuto R. (2018). Mitochondrial calcium uptake in organ physiology: From molecular mechanism to animal models. Pflügers Arch.-Eur. J. Physiol..

[19] Calvo-Rodriguez M., Bacskai B.J. (2020). High mitochondrial calcium levels precede neuronal death in vivo in Alzheimer’s disease. Cell Stress.

[20] Zhivotovsky B., Orrenius S. (2011). Calcium and cell death mechanisms: A perspective from the cell death community. Cell Calcium.

[21] Silberstein S.D., McCrory D.C. (2003). Ergotamine and dihydroergotamine: History, pharmacology, and efficacy. Headache J. Head Face Pain.

[22] Tfelt-Hansen P., Saxena P.R., Dahlöf C., Pascual J., Láinez M., Henry P., Diener H.-C., Schoenen J., Ferrari M.D., Goadsby P.J. (2000). Ergotamine in the acute treatment of migraine: A review and European consensus. Brain.

[23] Lee S., Seol H.-S., Eom S., Lee J., Kim C., Park J.-H., Kim T.-H., Lee J.H. (2022). Hydroxy Pentacyclic Triterpene Acid, Kaempferol, Inhibits the Human 5-Hydroxytryptamine Type 3A Receptor Activity. Int. J. Mol. Sci..

[24] Manoharan S., Guillemin G.J., Abiramasundari R.S., Essa M.M., Akbar M., Akbar M.D. (2016). The role of reactive oxygen species in the pathogenesis of Alzheimer’s disease, Parkinson’s disease, and Huntington’s disease: A mini review. Oxidative Med. Cell. Longev..

[25] Behl C., Moosmann B. (2002). Antioxidant neuroprotection in Alzheimer’s disease as preventive and therapeutic approach. Free. Radic. Biol. Med..

[26] Teleanu R.I., Chircov C., Grumezescu A.M., Volceanov A., Teleanu D.M. (2019). Antioxidant therapies for neuroprotection—A review. J. Clin. Med..

[27] Geiszt M., Leto T.L. (2004). The Nox family of NAD (P) H oxidases: Host defense and beyond. J. Biol. Chem..

[28] Poole L.B., Nelson K.J. (2008). Discovering mechanisms of signaling-mediated cysteine oxidation. Curr. Opin. Chem. Biol..

[29] Beal M.F. (1995). Aging, energy, and oxidative stress in neurodegenerative diseases. Ann. Neurol..

[30] Tramutola A., Lanzillotta C., Perluigi M., Butterfield D.A. (2017). Oxidative stress, protein modification and Alzheimer disease. Brain Res. Bull..

[31] Rauner C., Köhr G. (2011). Triheteromeric NR1/NR2A/NR2B receptors constitute the major N-methyl-D-aspartate receptor population in adult hippocampal synapses. J. Biol. Chem..

[32] Tovar K.R., McGinley M.J., Westbrook G.L. (2013). Triheteromeric NMDA receptors at hippocampal synapses. J. Neurosci..

[33] Sheng M., Cummings J., Roldan L.A., Jan Y.N., Jan L.Y. (1994). Changing subunit composition of heteromeric NMDA receptors during development of rat cortex. Nature.

[34] Cull-Candy S., Brickley S., Farrant M. (2001). NMDA receptor subunits: Diversity, development and disease. Curr. Opin. Neurobiol..

[35] Kumar A. (2015). NMDA receptor function during senescence: Implication on cognitive performance. Front. Neurosci..

[36] Guidi M., Kumar A., Foster T.C. (2015). Impaired attention and synaptic senescence of the prefrontal cortex involves redox regulation of NMDA receptors. J. Neurosci..

[37] Guidi M., Rani A., Karic S., Severance B., Kumar A., Foster T.C. (2015). Contribution of N-methyl-D-aspartate receptors to attention and episodic spatial memory during senescence. Neurobiol. Learn. Mem..

[38] Liu J., Chang L., Song Y., Li H., Wu Y. (2019). The role of NMDA receptors in Alzheimer’s disease. Front. Neurosci..

[39] Hallett P.J., Standaert D.G. (2004). Rationale for and use of NMDA receptor antagonists in Parkinson’s disease. Pharmacol. Ther..

[40] Collingridge G. (1987). The role of NMDA receptors in learning and memory. Nature.

[41] Gray J.A., Shi Y., Usui H., During M.J., Sakimura K., Nicoll R.A. (2011). Distinct modes of AMPA receptor suppression at developing synapses by GluN2A and GluN2B: Single-cell NMDA receptor subunit deletion in vivo. Neuron.

[42] Kochlamazashvili G., Bukalo O., Senkov O., Salmen B., Gerardy-Schahn R., Engel A.K., Schachner M., Dityatev A. (2012). Restoration of synaptic plasticity and learning in young and aged NCAM-deficient mice by enhancing neurotransmission mediated by GluN2A-containing NMDA receptors. J. Neurosci..

[43] Dumas T.C. (2005). Developmental regulation of cognitive abilities: Modified composition of a molecular switch turns on associative learning. Prog. Neurobiol..

[44] Sun Y., Cheng X., Hu J., Gao Z. (2018). The role of GluN2A in cerebral ischemia: Promoting neuron death and survival in the early stage and thereafter. Mol. Neurobiol..

[45] Hafenbreidel M., Todd C.R., Mueller D. (2017). Infralimbic GluN2A-containing NMDA receptors modulate reconsolidation of cocaine self-administration memory. Neuropsychopharmacology.

[46] Mulac D., Hüwel S., Galla H.-J., Humpf H.-U. (2012). Permeability of ergot alkaloids across the blood-brain barrier in vitro and influence on the barrier integrity. Mol. Nutr. Food Res..

